# Next Generation Probiotics for Neutralizing Obesogenic Effects: Taxa Culturing Searching Strategies

**DOI:** 10.3390/nu13051617

**Published:** 2021-05-12

**Authors:** Ana López-Moreno, Inmaculada Acuña, Alfonso Torres-Sánchez, Ángel Ruiz-Moreno, Klara Cerk, Ana Rivas, Antonio Suárez, Mercedes Monteoliva-Sánchez, Margarita Aguilera

**Affiliations:** 1 Department of Microbiology, Faculty of Pharmacy, Campus of Cartuja, University of Granada, 18071 Granada, Spain; angel_trm_@hotmail.com (Á.R.-M.); klara.cerk@gmail.com (K.C.); mmonteol@ugr.es (M.M.-S.); 2 Center of Biomedical Research, Institute of Nutrition and Food Technology “José Mataix”, University of Granada, Armilla, 18016 Granada, Spain; iacuna@ugr.es (I.A.); asuarez@ugr.es (A.S.); 3 Department of Biochemistry and Molecular Biology, Faculty of Pharmacy, Campus of Cartuja, University of Granada, 18071 Granada, Spain; 4 IBS, Instituto de Investigación Biosanitaria, 18012 Granada, Spain; amrivas@ugr.es; 5 Department of Nutrition and Food Science, Campus of Cartuja, University of Granada, 18071 Granada, Spain

**Keywords:** next-generation probiotics, culturing, dietary obesogens exposure, obesity, endocrine pathogenesis, Endobolome

## Abstract

The combination of diet, lifestyle, and the exposure to food obesogens categorized into “microbiota disrupting chemicals” (MDC) could determine obesogenic-related dysbiosis and modify the microbiota diversity that impacts on individual health–disease balances, inducing altered pathogenesis phenotypes. Specific, complementary, and combined treatments are needed to face these altered microbial patterns and the specific misbalances triggered. In this sense, searching for next-generation beneficial microbes or next-generation probiotics (NGP) by microbiota culturing, and focusing on their demonstrated, extensive scope and well-defined functions could contribute to counteracting and repairing the effects of obesogens. Therefore, this review presents a perspective through compiling information and key strategies for directed searching and culturing of NGP that could be administered for obesity and endocrine-related dysbiosis by (i) observing the differential abundance of specific microbiota taxa in obesity-related patients and analyzing their functional roles, (ii) developing microbiota-directed strategies for culturing these taxa groups, and (iii) applying the successful compiled criteria from recent NGP clinical studies. New isolated or cultivable microorganisms from healthy gut microbiota specifically related to obesogens’ neutralization effects might be used as an NGP single strain or in consortia, both presenting functions and the ability to palliate metabolic-related disorders. Identification of holistic approaches for searching and using potential NGP, key aspects, the bias, gaps, and proposals of solutions are also considered in this review.

## 1. Introduction

### 1.1. Microbiota Gut Dysbiosis

The microbiota is a microbial community that lives on and in the human body and it varies according to several factors such as age, diet, and lifestyle [1]. These microorganisms play a very important role in maintaining the health homeostasis or eubiosis [2]. It has been well-demonstrated that gastrointestinal tract (GIT) disorders are linked to microbiota alterations patterns (such as constipation, diarrhea, inflammatory bowel diseases [3,4]) that can be treated with probiotics. Moreover, important metabolic disorders, presenting altered levels of triacylglycerols, lipids, cholesterol, and fasting plasma glucose as clinical outcomes [5] are also linked to GIT dysbiosis. Similarly, fertility disorders such as polycystic ovary syndrome (PCOS) [6], gastrointestinal and reproductive cancers [7], or mental health disorders like depression, anorexia, or anxiety are also connected to microbiota dysbiosis [8].

### 1.2. Traditional Probiotics vs. NGP in Obesity-Related Interventions and Treatments

Probiotics, known as “live microorganisms, which, when administered in adequate amounts confer a health benefit on the host” by the Food and Agriculture Organization of the United Nations (FAO) and the World Health Organization (WHO) [9], have been empirically selected due to their extensive use in fermented foods for centuries and their safety history. Conversely, because of this broad definition, their use has become widespread, making them less effective against specific diseases [10]. Since then, numerous studies have been published in order to demonstrate the benefits of probiotics in an extensive list of disorders and/or diseases, traditional probiotics corresponding to strains or species generally within *Lactobacillus* and *Bifidobacterium* genera, and a few from other genera [11]. Traditional probiotics for clinical interventions in obesity-related disorders have been largely used, with huge differential impact on the clinical parameters and outcomes, depending on the basis of the individual microbiota (Table 1).

Additionally, it is well-known that the functional and specific positive biological effects of probiotics are strain-dependent. Therefore, validated clinical studies should define well the specific strains administered to the subjects as shown in Table 1 [12,13].

However, new advances in high-throughput and -omics technologies allowed scientific community to characterize and identify new microorganisms called next generation probiotics (NGP) according to the beneficial basic definition of a probiotic, but they are better characterized by targeting specific diseases and clinical outcomes. NGPs have been initially well-designed and tested for obesity-related disorders (Table 2). Moreover, according to O’Toole et al. [14], there are substantial differences in the way of investigating traditional probiotics vs. NGP, driven by the high-throughput current technologies available and cumulated data evidence. Traditional probiotics harbor a limited number of microbial genera and species and they were initially selected according to their long history of safe use. Also, these probiotics tend to be searched and marketed by companies targeting general, narrowly defined populations. While NGPs belong to a wide range of genera and species, they are investigated by multidisciplinary approaches with microbiome and clinical expertise, the main goal of which is to obtain effective biosources to palliate specific microbiota dysbiosis and associated phenotypic disorders.

**Table 1:** Traditional probiotics for obesity-related interventional clinical trials and preclinical studies.

*Lactobacillus* Strains [15]	Study Design, Target Species	Reference Study
*L. bulgaricus* Nutricion Medica^®^	ICT—Human	[16]
*L. casei* Shirota	ICT—Human	[17]
*L. gasseri* BNR17	ICT—Human	[18]
*L. reuteri* V3401	ICT—Human	[19]
*L. rhamnosus* CGMCC1.3724	ICT—Human	[20]
*L. acidophilus* NS1	PCS—Mice	[21]
*L. johnsonii* JNU3402	PCS—Mice	[22]
*L. plantarum* Ln4	PCS—Mice	[23]
*L.curvatus* HY7601	PCS—Mice	[24]
*L. fermentum* CQPC07	PCS—Mice	[25]
***Bifidobacterium* strains**	**Study design, Target Species,**	**Reference study**
*B. animalis* subsp. *lactis* 420	ICT—Human	[26]
*B. breve* B-3	ICT—Human	[27]
*B. infantis* DSM24737 (VSL#3)	ICT—Human	[28]
*B. lactis* HN019	ICT—Human	[29]
*B. longum* APC1472	ICT–Human/PCS–Mice	[30]
*B. adolescentis*	PCS—Mice	[31]
*B. bifidum* BGN4	PCS—Mice	[32]
***Bacillus, Enterococcus, Streptococcus* strains**	**Study design, Target Species,**	**Reference study**
*Bacillus coagulans* Unique IS2	ICT—Human	[33]
*Bacillus amyloliquefaciens* SC06	PCS—Mice	[34]
*Bacillus* spp.	PCS—Mice	[35]
*Enterococcus faecium* R0026	PCS—Mice	[36]
*Enterococcus faecalis* AG5	PCS—Rats	[37]
*Streptococcus thermophiles* MN-ZLW-002	PCS—Mice	[38]
***Saccharomyces* strains**	**Study design, Target Species,**	**Reference study**
*S. boulardii* Biocodex	PCS–Mice	[39]
*S. cerevisiae* SFBE	PCS–Rats	[40]

Traditional probiotics strains with obesity and anti-obesity effects. ICT: interventional clinical trials; PCS: preclinical studies.

## 2. Information and Criteria for Searching and Culturing Next-Generation Probiotics

**Figure 1:**
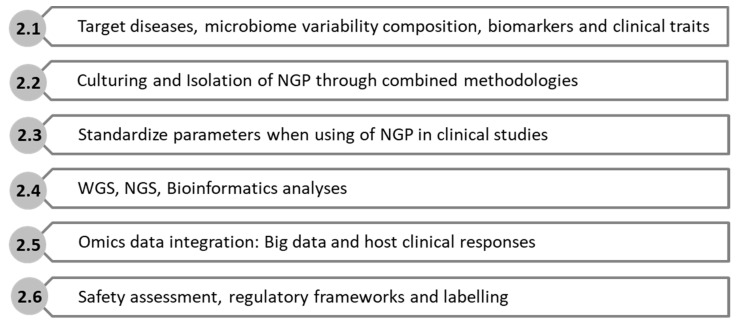
Next Generation Probiotics (NGP) criteria to be applied for searching strategies, Whole Genome Sequencign (WGS), Next Generation Sequencing (NGS):

The search for NGP that are able to modulate the effects of obesogenic and microbiota disruptor chemicals will request the following information according to the corresponding stepwise criteria (Figure 1).

### 2.1. Target Diseases, Microbiome Variability Composition, Biomarkers and Clinical Traits

#### 2.1.1. Obesity, Metabolic, and Endocrine Diseases: Variability of Microbiota Composition

Interestingly, multiple convergent clinical studies have found differences between the microbiota of obese and healthy patients [59]. The clearest biomarker related to obesity appears to be Firmicutes-to-Bacteroidetes ratio. A higher ratio has been observed in obese or metabolic syndrome populations compared to the healthy ones [60,61]. Specific taxa seem to contribute to this ratio in obese patients: the genera *Staphylococcus* [62,63] and *Clostridium* [64], inside the Firmicutes phylum, have been shown to have a positive association with obesity. Moreover, an increase in butyrate and acetate synthesis may contribute to an increase in energy harvest in obese people, and many butyrate-producing species belong to the Firmicutes phylum [65]. 

The main variations of microbiota taxa found in patients suffering from obesity, diabetes, metabolic syndrome, liver diseases, and endocrine-related disorders are summarized in Table 3. The present work focused on those species or taxa whose abundance was comparatively different between patients and healthy individuals. Therefore, isolating and culturing these microbial species would allow us to test and verify their biological functions, and if the effects were clinically proved, they could be proposed as beneficial microbial or NGP. 

Interestingly, levels of traditional probiotics from the genera *Lactobacillus* and *Bifidobacterium* seem to be higher in obesity- and endocrine-related diseases accordint to data retrieved and summarized in Table 3. Conversely, the species of NGP that are recognized and clinically tested, seem to be lower in obesity-related patients. Therefore, species tested from the genera *Akkermansia*, *Faecalibacterium*, *Eubacterium*, *Bacteroides*, *Parabacteroides*, and *Christensenella* could contribute to restore the microbial misbalances observed. In this sense, new beneficial microbes or NGP searching approaches might be successfully based on culturing and isolating those new genera and species that present a differential abundance between patients and healthy subjects and they can be linked to relevant clinical outcome.

#### 2.1.2. Nutrition and Diets, Dietary Exposure to Obesogens, and Microbiome Interactions

Dietary intake is considered one of the determining factors that modulate the microbial composition and diversity of the gut microbiome, which could promote either beneficial or negative effects on host health and physiological functions [92,93]. A Western-style diet, rich in animal-based foods, can increase the patient’s levels of bile-tolerant bacteria, including Bacteroidetes (e.g., *Bacteroides* and *Alistipes*), and Proteobacteria (*Bilophila*), and decrease levels of fiber-degrading bacteria such as Firmicutes (e.g., *Eubacterium* and *Ruminococccus*) [94]. Conversely, the Mediterranean diet and plant-based diets can promote fiber-degrading bacteria, mainly including genera of the Firmicutes phylum, together with increased overall diversity of the gut microbiota [95]. There are fewer studies about the associations between dietary habits and the gut microbiota in the Asiatic populations [96,97], which are characterized by higher intakes of several fermented foods containing microorganisms similar to probiotic strains [98,99], which could affect the composition and diversity of the gut microbiota, thus affecting human health [100]. 

In addition, globalized population has incorporated much more processed foods and artificial products into their diets to keep up with the rapid pace of lifestyles. Therefore, the exposure to dietary contaminants became a cause of health concern worldwide [101,102,103]. Processed foods could contain obesogens derived from endocrine-disrupting chemicals that have also an effect on the gut microbiota, promoting adipogenesis and weight gain, as well as microbiome dysbiosis [104,105], which is linked to multiple diseases and adverse health outcomes [106,107]. The enzymatic arsenal of gut microbiota plays a key role in metabolizing dietary obesogens from processed or cooked food, promoting different outcomes: (i) Gut microbiota could protect against the carcinogenic and genotoxic substances by degrading or biotransforming them to less toxic compounds or facilitating their excretion [108,109]. (ii) Gut microbiota may also detoxify xenobiotics, for example, into genotoxins, or may reverse the detoxification implied by the host metabolism [110]. (iii) Gut microbiota is capable of transforming xenobiotics into less toxic and mutagenic substances, thus it may be able to lessen the chances of cancer and other dysbiosis effects [111]. (iv) Gut microbiome (human/animals) might be negatively affected by several food/feed additives (sweeteners, emulsifiers, preservatives, etc.) and other contaminants (BPA, Parabens, Pesticides, etc.) through triggering microbiota dysbiosis. Consequently, advances in toxicomicrobiomics are needed to study these complex and mutual influences between the ever-changing microbiome and obesogens of various origins, with emphasis on their fate and toxicity, and xenobiotic-modifying enzymes [112].

### 2.2. Culturing and Isolation of NGP through Combined Methodologies

The search for microbiological differences between the study groups (such as the healthy and the dysbiotic taxa groups) allows us to identify potential probiotics, and even detoxifying microorganisms, which could be used as NGP. However, this is followed by isolation and characterization of potential probiotics, and so far, none of the bacteria in the microbiota can be cultured in vitro yet [113]. This could be due to the difficulties of replicating essential aspects of their anaerobic environment [114] or the need to coculture with other bacteria from the same environment [115]. However, new media and modified procedures, such as improved culturomics, are continuously developing and evolving. They consist of multiple culture conditions with rapid identification of bacteria, raising the level of cultured bacteria and their possible use as bioresources or even NGP [116]. Table 4 summarizes the main putative new species isolated from recent culturing approaches in connection with the highlighted species underrepresented in obesity, which could be restored by a supplemented formula. Moreover, the isolation of strains from human microbiota able to biodegrade xenobiotics is successful through a directed cultivation approach with enriched media containing the specific xenobiotic [117]. BPA-tolerant strains were isolated in 30% of infant fecal microbial culture libraries analyzed. Most isolated strains were phylogenetically related to the operational taxonomic group *Bacillus amyloliquefaciens.* The culture media most used for cultivation of specific gut microbial strains with success were yeast-extract-casein hydrolysate-fatty acids (YCFA); gifu anaerobic medium (GAM); brain–heart infusion (BHI); eosin methylene blue (EMB); Lactobacillus selection (LBS); gut microbiota medium (GMM); and Man, Rogosa, and Sharpe (MRS).

### 2.3. Standardize Parameters When Using NGP in Clinical Studies

Traditional probiotics (Table 1) were not regulated as drugs but instead as dietary supplements; they are not subjected to the same rigorous standards and could have quality control issues [124]. As previously described, numerous studies have been carried out to prove the benefits of probiotics in a large number of dysbioses, but without standardized steps on dosages, patterns of administration, and detailed strains. 

There is no consensus on the minimum number of microorganisms that should be ingested to obtain a beneficial effect [125]. Since the effective dose of probiotics is influenced by multiple variables, it is difficult to standardize an optical dose [126]. Additionally, there is a need to investigate potential synergistic effects or antagonistic activity between strains in multi-strain vs. single-strain products [127]. Furthermore, it is well- demonstrated that the positive biological effects that the probiotics exert are strain-dependent, so it is necessary to obtain a taxonomic characterization to the strain level [12,13]. In previous reviews [128,129], we have seen an unharmonized broad range of intervention, total dose, and administration patterns of probiotics in obesity and fertility disorders. Finally, another parameter to be harmonized is the target population, since it has been seen that the beneficial effect of a probiotic in a population may not be adequate for another population, even causing potential adverse effects [130].

### 2.4. Whole Genome Sequencing, Next-Generation Sequencing, and Bioinformatics Analyses

The rapid evolution of cultivation-independent, next-generation sequencing, and meta-omics technologies has allowed for the integration and analyses of large datasets for the study of the diversity, complexity, and functional role of the human gut microbiome in health and disease [131]. A large part of the detected bacteria has never been cultivated [132]. Therefore, an integrative approach using both metagenome and metabolome-based characterizations of the gut microbiome together with bioinformatics and statistical filters and algorithms can provide strain-level taxonomic resolution of the taxa present in microbiomes, assess the potential functions encoded by the microbial community and quantify the metabolic activities within a complex microbiome [133]. 

**Figure 2:**
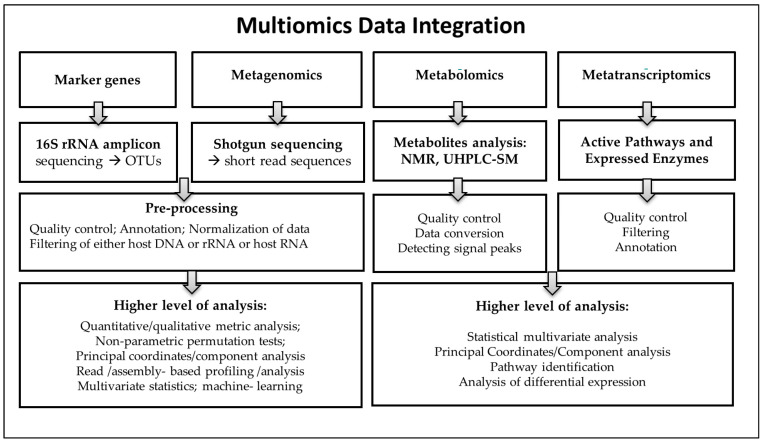
Multiomics and bioinformatics analysis of microbiome components.

The various platforms and reference databases developed for the marker gene (16S rRNA), metagenomics, or metatranscriptomics analysis often use similar stepwise approaches (Figure 2) with different bioinformatic tools (DADA2, Deblur, Kraken, MEGAN, HUMAnN, metaSPAdes, MEGAHIT, QIIME, Mothur, and several R packages (vegan, microbiome, etc.). 

### 2.5. Omics Data Integration: Big Data and Host Clinical Responses

As previously mentioned, microbiomics give us a great insight into the regulation of gut microbiota. However, in order to understand the complex biological pathways behind diseases, the identification of novel -omics biomarkers, such as identification of genes (genomics), gene expressions and phenotype (epigenomics), messenger RNA and micro RNA (transcriptomics), proteins (proteomics), and metabolites (metabolomics, lipidomics, glycomics) could bring forward knowledge on probiotics and their effects on obesity and its modulation of pathophysiological mechanisms that have links with chronic diseases [134,135]. 

Integrating multi-omics datasets is an innovative assignment, due to the increased complexity and diversity of the collected data [136]. This integration is increasingly reliant on efficient bioinformatics tools and advanced statistical methods [137,138,139]. Multi-omics data integration still poses challenges, but integration of multiple meta-omics datasets lays out a promising approach to comprehensively characterizing the composition, functional, and metabolic activity of microbiomes. This is of particular importance for microbiome research to be translated into clinical applications and further improvement of human health management [140].

### 2.6. Safety Assessment, Regulatory Frameworks, and Market Labeling

The overview of worldwide regulatory frameworks affecting different food categories is summarized in Table 5. 

Overall, in the European Union (EU), most bacteria that will be used in foods for human consumption need to comply with two different regulations [141,142], or if used as life biotherapeutic products, as clarified in the European Pharmacopoeia (Ph. Eur.) [143]. At the same time, in the US, probiotics should be classified as microorganisms with a qualification of “generally recognized as safe” (GRAS) by the Food and Drug Administration (FDA). Both regulatory frameworks largely involve scientific requirements [14]. Furthermore, in order to assess the safety of microorganisms, the European Food Safety Authority (EFSA) introduced the concept of qualified presumption of safety (QPS) to harmonize the safety evaluation of microorganisms used as food or feed additives, food enzymes, novel foods, or pesticides, which has to follow certain criteria [144]. 

**Table 5:** Summary of probiotics categorization and regulation frameworks worldwide.

Country	Category	Regulatory Framework	Claims	Reference
**USA**	Drugs, nutraceuticals	FDA	Health claims Nutrient claims Structure claims GRAS	[145,146]
	Dietary supplements	DSHEA	Probiotics considered as foods		
	Biological product	FDA (BLA)	Probiotics as a reference product, biosimilar product, or an interchangeable product; solely to be used for medical therapeutic purpose		
	Life biotherapeutic agent	FDA	Probiotics as a biological product that contains live organisms and is applicable to the prevention, treatment, or cure of a disease or condition; recombinant life biotherapeutic agent		
	Medical Food	FDA/DSHA	Probiotics specially formulated to be intended for dietary management under supervision; medical foods are exempt from the labeling requirements for nutrient content and health claims		
**China**	Functional foods	SFDA	Conventional foods mark (the presence of a specific ingredient in the label of regular foodstuffs) Healthy foods (the presence of health function)	[147]
**Europe**	Functional Food and nutraceuticals	EFSA (FUFOSE)	Health claims, nutrition claims QPS	[143,144,148]
	Life biotherapeutic products	EMA	Probiotics as medicinal products containing live microorganisms for human use	
**Japan**	Functional foods and nutraceuticals	MHLW, FOSHU	Foods with functional claims Foods with nutrient functional claims	[149,150]
**Canada**	Natural health products	FDA (CFIA)	Nutrient content claims Health claims	[151]

EFSA: European Food Safety Agency; EMA: European Medicines Agency; FAO/WHO: Food and Agricultural Organization/World Health Organization; MHLW: Ministry of Health and Welfare; FOSHU: food for specified health use; FUFOSE: functional food science in Europe; SFDA: State Food and Drug Administration; DSHEA: Dietary Supplement Health and Education Act; BLA: biologic license application; CFIA: the Canadian Food Inspection Agency.

However, despite all preventive effects, the consumption of probiotics may not be completely safe in certain cases or physiological states [14]. In this context, several bacterial species comprising genera other than *Lactobacillus* and *Bifidobacterium* with proven efficacy, which are considered as potential NGP, may be strain-by-strain assessed in order to obtain sufficient research data, and to grant probiotic status on the species and strain levels [152].

Information of beneficial results provided by the NGP will encompass comprehensive understanding of their targeted diseases. On top of these, the underlying molecular mechanisms on how NGP work and interact with the host have to be clarified [153]. It is important to characterize in vitro bacterial physiology, genomic analysis of potential virulence and antimicrobial resistance genes, investigations on the presence or absence of potential genes involved in transferring antibiotic resistance gene, and in vivo acute toxicity studies in both healthy and immunosuppressed mice [154].

The regulation of marketed probiotics applies differently among countries according to their classifications, and the country’s nutritional and dietary habits and lifestyle. Therefore, probiotics can be classified as nutraceuticals, dietary supplements, or food. Regulation and requirements for the safety assessment of beneficial microbes is variable within countries [155,156,157,158]. Probiotics, food supplements, labeling and other information to consumers are regulated under the legislation [159,160]. On the opposite side, the US and its FDA, responsible for quality control of probiotics, has taken the approach of having minimal regulation [161]. Most probiotic products in the US are classified as food or dietary supplements, which have to comply with good manufacturing practice (GMP) guidelines [162]. Harmonization and consensus of all stakeholders involved in the probiotic market could be important since boundaries between differently regulated markets have become minimal [144]. 

Therefore, next-generation beneficial microbes’ approval procedures should be enforced according to their classifications [154,155,156,157,158,159], stating the general safety of the product and using harmonized descriptions: the genus, species, and strains used, the CFU/g or mL of product (colony-forming units), the recommended use, and the daily dose; as well as quality and market parameters of the products: trademarks, formulae, ingredients, expiration dates, and storage conditions [151].

## 3. Discussion

The use of fermented food containing beneficial microbes is an ancestral tradition. Moreover, classical probiotics have been administered in several disorders and also specifically in obesity and metabolic diseases. However, they do not always provide harmonized endpoints data [136]. Controversial results have triggered the continuous need for searching and elucidating how to better understand and optimize the use and consumption information of probiotics. The combined impact of differential diets and the complementary probiotic strains should be standardized according to the individual and their microbiota composition and status [130]. Moreover, tested administration patterns and robust evidence of probiotics’ clinical beneficial impact should be well-supported by clinical trials [14]. 

Therefore, NGP as well as the described new beneficial microbial species and strains [10] constitute a growing trend of searching for biotechnological uses. NGP could be considered as a complementary, preventive and/or therapeutic tool for standardized interventional clinical studies [48,49]. However, NGP searching strategies, culturing research, and clinical implementation still face challenges, and there are specific gaps to be covered regarding bioinformatics and statistical analysis, safety assessment, specific strains, and the frame regulation on marketing and labeling [145,146,147,148]. Regarding the bioinformatics analysis, the limitations are related to the capabilities of the different platforms used. Statistical analysis faced problems of high dimensionality, over-dispersion, sparsity, and zero-inflation of data. Safety assessments lack proven efficacy at species level (in vitro test; genomic analysis for identifying potential virulence and antimicrobial resistance genes; in vivo acute toxicity tests), while the regulations frame lacks global harmonization and consensus from all stakeholders involved in the probiotics market, together with clear, reliable, and truthful labeling, focusing specifically on the level of genus, species, and strain used in the product. The label should clearly state the genus, species, and strain used, CFU/g or mL of product (colony-forming units), and the recommended use and daily dose. Moreover, it should refer to the quality parameters and market conditions [151].

More standardization efforts and research intervention strategies should focus on modulatory microbiota capacities and envisage the development and use of NGP, the formulation of which requires competent preclinical studies to show their efficacy and safety status. In overall terms, such advances and directions could help researchers, clinicians, dietitians, and nutritionists in using harmonized probiotics supplementary recommendations and targeted effects. Moreover, a joint effort to incentivize the reuse of published clinical data as open access (OA) [163] will make available more data for robust comparisons.

Next-generation probiotics are emerging microorganisms with demonstrated clinical impact, well-defined modes of actions, and specific functions impacting target diseases. The microbiota of healthy individuals appeared enriched in microorganisms considered NGP such as *A. muciniphila, F. prausnitzii, Eubacterium* spp., within other several species that seem to contribute to a balanced intestinal microbiota [48,49]. Interestingly, these same species were lower in obesity-related disorders. Thus, the present work has focused on searching and culturing approaches for other profiled and decreased levels of microbial species in metabolic diseases. 

Specific approaches for obtaining specific NGP that neutralize dietary obesogens and their effects have been discussed. 

## 4. Conclusions

Therefore, the present work highlights the taxa culturing pathways and key topics for extrapolating and aligning investigation efforts on searching for NGP to target diseases where the interventional modulation studies of microbiota impact on health status. The present work allowed us to highlight the following needs and conclusions:

- Culturing of microorganisms from microbiota is the key activity to obtain NGP from healthy individuals, mainly through isolating those microorganisms identified as differentially decreased in the target disease or abundant in healthy microbiota, focusing on candidatus species from metagenomics studies.
- Screening and selection of the potential NGP in a target-disease population by using in vitro models before clinical interventions.
- Harmonization on performing exhaustive pre-analysis and post-intervention of individual microbiota composition through representative and validated methodologies (e.g., V3–V4 and Illumina MiSeq technology) is needed before administering NGP.
- There is a need to standardize bioinformatics and database tools for specifically designing analysis of large and universal microbiome datasets.
- NGP single strains or taxa consortium should have attributable documented benefits and their safety confirmation statements.
- Effective doses and well-defined patterns of administration of NGP should become factors for aligning intervention doses since the beginning of clinical translation.
- International guidelines on NGP and microbiota investigations for targeting obesity-related diseases prevention or treatments are needed. This will allow for more meaningful effect comparisons of harmonized and valuable studies, facilitating more robust meta-analysis.
- Data reuse and availability of open access interventional clinical trials data will contribute to obtaining significant association of clinical outcomes.

## Figures and Tables

**Table 2:** Next-generation probiotic strains used in obesity-related clinical trials and preclinical studies.

NGP Microbial Strains, Target Species, Study Reference	Study Design	Dietary Aspects	Clinical Effects and Functionality
*Akkermansia muciniphila* Muc [CIP 107961]—Human [41] [ClinicalTrials.gov Identifier: NCT02637115]	ICT: randomized, double-blind, placebo-controlled pilot study Live probiotics 10^10^/day vs. pasteurized probiotics 10^10^/day vs. placebo in patients with metabolic syndrome	Normal dietary intake and physical activity during the study period	↑ Insulin sensitivity, ↓ insulinemia and ↓plasma total cholesterol
*Akkermansia muciniphila* WST01—Human [42] [ClinicalTrials.gov Identifier: NCT04797442]	ICT: randomized, double-blind, placebo-controlled, multicenter clinical trial Probiotics vs. placebo in overweight or obese patients with type 2 diabetes	Intervention added onto lifestyle	Results will be available in June 2022
*Christensenella minuta* Xla1—Human [43] [ClinicalTrials.gov Identifier: NCT04663139]	ICT: randomized, partially placebo-controlled double-blind Probiotics vs. placebo in healthy volunteers, overweight, and obese adults	Agreement to keep food, drink, physical activities, and alcohol consumption habits unchanged throughout the study	Results will be available in October 2021
*Eubacterium hallii*—Human [44] [ClinicalTrials.gov Identifier: NCT04529473]	ICT:double-blind, randomized, placebo-controlled Probiotics vs. placebo	Maintenance of dietary habits and physical activity levels throughout the study period	Results will be available on January 2022
*Hafnia alvei* HA4597—Human [45] [ClinicalTrials.gov Identifier: NCT03657186]	ICT: multicenter, randomized, double-blind placebo-controlled study. Probiotics vs. placebo on weight reduction in overweight subjects	−20% hypocaloric diet and maintainance of the usual physical activity	↑ Weight loss in overweight subjects, ↑ feeling of fullness, ↑ loss of hip circumference, ↓ fasting glycemia
*Lactococcus lactis* NRRL-B50571—Human [46] [ClinicalTrials.gov Identifier: NCT02670811]	ICT: double-blind randomized controlled Probiotics vs. placebo on prehypertensive subjects	Participants were asked not to change their diet or lifestyle during the intervention	↓ Systolic and diastolic blood pressure, ↓ Triglyceride, total cholesterol, and low-density lipoprotein
*Escherichia coli* Nissle 1917—Human [47] [ClinicalTrials.gov Identifier: NCT02144948]	ICT: single group assignment. Patients with type 2 diabetes	-	Results not yet available or posted on ClinicalTrials.gov November 2021
*Akkermansia muciniphila*—Muc [CIP 107961]—Mice [48,49]	PCS: probiotics vs. control. Obesity	High-fat diet/standard diet	↓ Fat-mass gain, ↑ insulin sensitivity, restore gut barrier function by acting on TLR2, ↑ mucus later thickness; similar effects by a purified membrane protein alone (Amuc_1100)
*Clostridium butyricum* CGMCC0313.1—Mice [50]	PCS: probiotics vs. control. Obesity	High-fat diet/standard diet	↓ Lipid accumulation in liver and serum, ↓ insulin levels, ↑ glucose tolerance, ↑ insulin sensitivity, ↓ TNF-α and ↑ IL-10 and IL-22 in colon
*Faecalibacterium prausnitzii* VPI C13-20-A—Mice [51]	PCS: probiotics vs. control. Obesity	High-fat diet/standard diet	↑ Hepatic health, ↓ adipose tissue inflammation
*Bacteroides uniformis* CECT 7771– Mice [52]	PCS: probiotics vs. control. Obesity	High-fat diet/standard diet	↓ Weight gain; ↓ dietary fat absorption; ↓ liver steatosis; ↓ serum cholesterol, triglyceride, glucose, insulin and leptin; ↑ glucose tolerance; ↑ TNF-α by DCs after LPS stimulation;↑ phagocytosis
*Parabacteroides goldsteinii* JCM 13446—Mice [53]	PCS: probiotics vs. control. Obesity	High-fat diet/standard diet	↓ Obesity by ↑ adipose tissue thermogenesis, ↑ intestinal integrity ↓ inflammation, ↑ insulin sensitivity
*Christensenella minuta*—Mice [54]	PCS: probiotics vs. control. Obesity	High-fat diet/standard diet	↓ Weight gain, ↓ adiposity. Highly heritable in a lean host phenotype
*Eubacterium hallii* DSM 17630—Mice [55]	PCS: probiotics vs. control. Diabetes	High-fat diet/standard diet	↑ Energy metabolism and ↑ insulin sensitivity through glycerol conversion 3hydroxypropionaldehyde
*Hafnia alvei* HA4597—Mice [56]	PCS: probiotics vs. control. Obesity	High-fat diet/standard diet	↑ Beneficial anti-obesity and metabolic effects, ↓ food intake, ↓ body weight and ↓ fat mass gain
*Lactococcus lactis* (GMM) LL-pCYT: HSP65-6P277 and LL-pHJ—Mice [57]	PCS: probiotics vs. control. Obesity	High-fat diet/standard diet	↓ Antigen-specific of cellular immunity
*Escherichia coli Nissle 1917* (EcN-GMM)– Mice [58]	PCS: probiotics vs. control. Obesity	High-fat diet/standard diet	Modulation of the neuropeptide expression of energy intake and expenditure in the hypothalamus

NGP tested with anti-obesity effects; DC: dendritic cells; IL: interleukin; ICT: interventional clinical trials; LPS: lipopolysaccharide; PCS: preclinical studies; TLR2: toll-like receptor 2; TNF: tumor necrosis factor.

**Table 3:** Clinical trials and variations of the main microbiota taxa found in specimens from patients suffering metabolic- and endocrine-related diseases.

Reference	Subjects and Disease	Dietary Aspects	Sample Size and Clinical Traits	Detection Technique	Microbial Taxa Modifications
Zhong et al. [66]	Human Obesity	NA	*N* = 382; MHNO *n* = 191; MUNO *n* = 61; MHO *n* = 66; MUO *n* = 64	MiSeq platform (Illumina) V3–V4 region of the 16S rRNA gene	↑ *Lachnospiraceae*, *Bacteroidaceae,* *Methanobacteriaceae* and *Pasteurellaceae* in MHNO and MUNO
Jonduo et al. [67]	Human Obesity	Participant’s predominantly plant-based diet: vegetables (e.g., sweet potato, cassava, plantain, and beans)	*n* = 18; OB *n* = 9; Non-OB *n* = 9	454 GS FLX platform or 454 GS JUNIOR system (Roche) V1-V2 region of the 16S rRNA gene	↑ *Prevotella* in almost all individuals
Thingholm et al. [68]	Human Obesity	NA	*n* = 1280; LH *n* = 633; OBH *n* = 494; OBT2D *n* = 153	MiSeq platform (Illumina) V1-V2 region of 16S rRNA gene	↓ ***Akkermansia***, ***Faecalibacterium***, ***Oscillibacter***, and ***Alistipes*** in obese individuals ↓ ***Faecalibacterium prausnitzii*** in obese individuals
Schwiertz et al. [65]	Human Obesity	Western diet	*n*= 98; HC *n* = 30; OW *n* = 35; OB *n* = 33	qPCR	↑ *Bacteroides* in overweight vs. HC ↓ ***Ruminococcus flavefaciens*** in overweight and obese ↓ ***Bifidobacterium*** and ***Clostridium leptum*** in obese ↓ ***Methanobrevibacter*** in overweight and obese
Gao et al. [69]	Human Obesity	NA	*n* = 192; HC *n* = 25; OW *n* = 22; OB *n* = 145	MiSeq platform (Illumina) V4 region of the 16S rRNA gene	↑ *Lachnoclostridium, Fusobacterium, Escherichia-Shigella, Klebsiella, Bacillus*, and *Pseudomonas* in OW and OB ↑ *Clostridia, Faecalibacterium, Ruminococcus, Bifidobacterium,* and *Lachnospiraceae_UCG_008* in HC
Armougom et al. [70]	Human Obesity Anorexia nervosa	NA	*n*= 49; HC *n* = 20; OB *n* = 20; AN *n* = 9	qPCR	↑ *Lactobacillus* in OB
Horie et al. [71]	Mice Type 2 diabetes	NA	5-week-old TSNO mice *n* = 5; 5-week-old TSOD mice *n* = 5; 12-week-old TSNO mice *n* = 5; 12-week-old TSOD mice *n* = 5	qPCR	↑ *Lactobacillus* in TSOD vs. TSNO ↑ *Bacteroidales* and *Lachnospiraceae* in TSNO vs. TSOD ↑ *Turicibacter* and SMB53 in TSOD
Larsen et al. [72]	Human Type 2 diabetes	NA	*n* = 36; HC *n* = 18; T2D *n* = 18	MiSeq platform (Illumina) V4 region of the 16S rRNA gene	↑ Firmicutes in HC ↑ Bacteroidetes and *Betaproteobacteria* in T2D ↓ **Clostridia** in T2D
Sedighi et al. [73]	Human Type 2 diabetes	NA	*n* = 36; HC *n* = 18; T2D *n* = 18	qPCR	↑ *Lactobacillus* in T2D ↑ *Bifidobacterium* in HC ↑ *Fusobacterium* in T2D
Moghadam et al. [74]	Human Tipe 2 diabetes	NA	*n* = 36; HC *n* = 18; T2D *n* = 18	qPCR	↑ *Faecalibacterium prausnitzii* in HC
Ahmad et al. [75]	Human Type 2 diabetes Obesity	Eastern dietary habits (high carbohydrate and fat intake, low fiber intake)	*n* = 60; HC *n* = 20; Obese-T2D *n* = 40	MiSeq platform (Illumina) V3–V4 region of the 16S rRNA gene	↑ Firmicutes in Obese-T2D ↑ Clostridia, Negativicutes, Coriobacteria*,* Acidobacteria*,* Deferribacteres, and Gemmatimonadetes in obese-T2D ↑ Verrucomicrobia, Bacteroidetes, Proteobacteria, and Elusimicrobia in HC ↑ *Prevotella* *P4_76,* Clostridiales*,* *Porphyromonadaceae* bacterium *DJF B175, Candidatus* *Alistipes marseilloanorexic* *AP11,* *Bacillus sporothermodurans,* *Staphylococcus* *SV3*, and *Iamia* in obese-T2D
Ejtahed et al. [76]	Human Type 2 diabetes Type 1 diabetes	NA	*n* = 110; HC *n* = 40; T2D *n* = 49; T1D *n* = 21	qPCR	↑ *Escherichia, Prevotella*, and *Lactobacillus* in T1D and T2D ↑ *Bifidobacterium, Roseburia*, and *Bacteroides* in HC ↓ ***Faecalibacterium*** in T1D vs. HC and T2D
Takagi et al. [77]	Human Type 2 diabetes Hypertension Hyperlipidemia	NA	*n* = 239; HC *n* = 54; HT *n* = 97; HL *n* = 96; T2D *n* = 162	MiSeq platform (Illumina) V3–V4 region of the 16S rRNA gene	↑ Actinobacteria in HT, HL, T2D, RISK2, and RISK3 ↓ **Bacteroidetes** in HT, HL, T2D and RISK3 ↑ *Bifidobacterium* in HL, T2D, RISK1 and RISK2 ↑ *Collinsella* in HT, HL, T2D, RISK2 and RISK3 ↑ *Escherichia* in RISK 3 ↓ ***Alistipes*** in HL
Wang et al. [78]	Human Non-alcoholic fatty liver disease	Omnivorous Chinese diet	*n* = 126; HC *n* = 83; NAFLD *n* = 43	454 Life Sciences Genome Sequencer FLX system (Roche) V3 region of the 16S rRNA gene	↓ Firmicutes ↑Bacteroidetes in NAFLD ↑ Bacteroidia ↓ **Clostridia** in NAFLD ↓ ***Coprococcus****, **Pseudobutyrivibrio**, **Moryella**, **Roseburia**, **Anaerotruncus**, **Ruminococcus**,* ***Anaerosporobacter***, and***Lactobacillus*** in NAFLD
Li et al. [79]	Human Non-alcoholic fatty liver disease	No dietary restrictions imposed	*n* = 67; HC *n* = 37; NAFLD *n* = 30	MiSeq platform (Illumina) V4 region of the16S rRNA gene	↑ *Lactobacillaceae,* *Peptostreptococcaceae, Veillonellaceae,* EtOH8*,* ***Coprobacillaceae***, and *Erysipelotrichaceae* in NAFLD ↑ *Porphyromonas and Succinivibrio* in NAFLD ↓ ***Odoribacter*** *and Proteus* in NAFLD
Shen et al. [80]	Human Non-alcoholic fatty liver disease	NA	*n* = 47; HC *n* = 22; NAFLD *n* = 25	454 GS-FLX platform (Roche) V3-V5 region of the 16S rRNA gene	↑ *Proteobacteria*, Fusobacteria, *Lachnospiraceae_*Incertae_Sedis and *Blautia* in NAFLD ↑ Bacteroidetes and *Prevotella* in HC ↑ *Escherichia_Shigella,* *Clostridium_XVIII*, and *Staphylococcus* in NAFLD
Raman et al. [81]	Human Non-alcoholic fatty liver disease	No dietary restrictions imposed	*n* = 60; HC *n* = 30; NAFLD *n* = 30	qPCR	↑ *Lactobacillus, Roseburia, Dorea*, and *Robinsoniella* in NAFLD ↓***Oscillibacter***in NAFLD
Michail et al. [82]	Human Non-alcoholic fatty liver disease Obesity	No dietary restrictions imposed	*n* = 50; HC *n* = 26; NAFLD *n* = 13; Obese non-NAFLD *n* = 11	qPCR	↑ *Gammaproteobacteria, Prevotella*, and *Epsilonproteobacteria* in NAFLD ↓ **Clostridia** ↑ *Alphaproteobacteria* in obese non-NAFLD
Nistal et al. [83]	Human Non-alcoholic fatty liver disease Obesity	NA	*n* = 73; HC *n* = 20; Obese-NAFLD *n* = 36; Obese non-NAFLD *n* = 17	MiSeq platform (Illumina) V3–V4 region of the 16S rRNA gene	↑ *Bacilli* in obese-NAFLD ↓ *Betaproteobacteria* in obese-NAFLD vs. obese non-NAFLD ↓ ***Oscillospira****, **Akkermansia***, and ***Eubacterium*** in obese-NAFLD and obese non-NAFLD vs. HC ↑ *Megasphaera, **Lactobacillus***, *Acidominococcus* in obese-NAFLD, and obese non-NAFLD vs. HC ↓ ***Blautia****, **Alkaliphilus***, and ***Flavobacterium*** in obese-NAFLD ↑ *Staphylococcus* in obese-NAFLD
Loomba et al. [84]	Human Non-alcoholic fatty liver disease Fibrosis	NA	*n*= 86; NAFLD *n* = 72; Fibrosis *n* = 14	qPCR	↑ Firmicutes in NAFLD, ↑ *Proteobacteria* in fibrosis ↑ *Eubacterium rectale* and *Bacteroides vulgatus* in NAFLD ↑ *Bacteroides vulgatus* and *Escherichia coli* in fibrosis ↓ ***Ruminococcus obeum****,* and ***Eubacterium rectale*** in fibrosis
Del Chierico et al. [85]	Human Non-alcoholic fatty liver disease Non-alcoholic steatohepatitis Obesity	NA	*n*= 115; HC *n* = 54, OB *n* = 8; NAFLD *n* = 27; NASH *n* = 26	454- Junior Genome Sequencer FLX system (Roche) V1-V3 region of the 16S rRNA gene	↑ *Bradyrhizobium*, *Anaerococcus*, *Peptoniphilus*, *Propionibacterium acnes, Dorea*, and *Ruminococcus* ↓ ***Oscillospira*** and ***Rikenellaceae*** in NAFLD ↑ *Ruminococcus, Dorea*, and *Blautia* in NASH
Da Silva et al. [86]	Human Non-alcoholic steatohepatitis Simple steatosis	7-day food record	*n* = 67; HC *n* = 28; SS *n* = 15: NASH *n* = 24	MiSeq platform (Illumina)	↓ ***Ruminococcus***, ***Faecalibacterium******prausnitzii***, and ***Coprococcus*** in NASH and SS vs. HC
Mouzaki et al. [87]	Human Non-alcoholic steatohepatitis Simple steatosis	HC patients were consuming more calories per kg compared to patients with NASH	*n* = 50; HC *n* = 17; SS *n* = 11; NASH *n* = 22	qPCR	↓ Bacteroidetes in NASH vs. SS and HC ↑ *Clostridium coccoides* in NASH vs. SS
Zhu et al. [88]	Human Non-alcoholic steatohepatitis Obesity	NA	*n*= 63; HC *n* = 16; OB *n* = 25; NASH *n* = 22	qPCR	↑ *Bacteroides* ↓ Firmicutes in NASH and OB ↓ ***Blautia*** and ***Faecalibacterium*** in NASH and OB
Boursier et al. [89]	Human Non-alcoholic steatohepatitis Fibrosis	NA	*n* = 57; Non-NASH *n* = 20 NASH *n* = 10; Fibrosis ≥ 2 *n* = 27	Illumina V4 region of 16S rRNA gene	↑ *Bacteroides* ↓***Prevotella*** in NASH ↑ *Bacteroides* and *Ruminococcus* in fibrosis ≥ 2 ↓ ***Prevotella*** in fibrosis ≥ 2
Qin et al. [90]	Human Cirrhosis	NA	*n*= 179; HC *n* = 83; Cirrhosis *n* = 96	qPCR	↑ *Streptococcus, Veillonella, Clostridium* and *Prevotella* in cirrhosis ↑ *Eubacterium* and *Alistipes* in HC ↓ ***Bacteroides*** in cirrhosis
Lim et al. [91]	Human Methabolic syndrome	NA	*n* = 655; Monozygotic twins *n* = 306; Dizygotic twins *n* = 74; Siblings *n* = 275	MiSeq platform (Illumina) V4 region of the 16S rRNA gene	↑ *Lactobacillus, Sutterella* and *Methanobrevibacter* in MetS ↓ ***Parabacteroides****, **Bifidobacterium**, **Odoribacter**, **Akkermansia*** and ***Christensenella*** in MetS

Genera and species in bold letters highlight the decreased microorganisms to be considered as potential NGP to be searched, cultured and assayed for their anti-obesity modulation effects. AN: anorexia nervosa; HC: healthy control; HL: hyperlipidemia; HT: hypertension; LH: lean healthy; MetS: metabolic syndrome; MHNO: metabolically healthy non-obese; MHO: metabolically healthy obese; MUNO: metabolically unhealthy non-obese; MUO: metabolically unhealthy obese; NA: Not applicable; NAFLD: non-alcoholic fatty liver disease; NASH: non-alcoholic steatohepatitis; OB: obese; OBH: obese healthy; OBT2D: obese type 2 diabetes; OW: overweight; RISK1: patients with only one disease; RISK2: patients with two diseases; RISK3: patients with three diseases; SS: simple steatosis; T1D: type 1 diabetes; T2D: type 2 diabetes; TSNO: Tsumura Suzuki Obese Diabetes mice; TSOD: Tsumura Suzuki, Non-Obesity mice.

**Table 4:** Culturing approaches to favor specific microbiota species and NGP taxa and candidatus *species.*

Reference/Sample	Culture Media	Culture Media Modifications	Selected Favored Cultured Microorganisms	Outcome and Observations: New Species Cultured: Potential NGP
Browne et al. [118] Human	**YCFA**	Glucose (0.2%), maltose (0.2%), and cellobiose (0.2%)	Aero-intolerant genus and species	68 new isolated species: 16S RNA similarity 86–97% ***Anaerotruncus*** *colihominis* ***Blautia*** *luti; B. hydrogenotrophica* ***Clostridium*** *boltae; C. celerecrescens; C. celerescens; C. clostridioforme; C. cocleatum; C. disporicum; C. ghonii; C. hathewayi; C. innocuum; C. lituseburense; C. methylpentosum; C. nexile; C. oroticum; C. saccharogumia; C. saccharolyticum; C. thermocellum; C. xylanolyticum* ***Coprococcus*** *eutactus* ***Oscillibacter*** *valericigenes* ***Roseburia*** *faecis; R. inulinivorans* ***Ruminococcus*** *albus; R.bromii; R. flavefaciens; R. gnavus*; *R.obeum; R. torques*
	**YCFA**	Pre-treatment with ethanol 70% (*v*/*v*), glucose (0.2%), maltose (0.2%), cellobiose (0.2%), sodium taurocholate (0.1%). Spore-forming gut aero-intolerant bacteria	***Alistipes** finegoldii* ***Anaerotruncus** colihominis * ***Blautia** hydrogenotrophica; B. obeum; B. wexlerae* ***Clostridum** baratti; C. bartlettii; C. clostridioforme; C. disporicum; C. hathewayi; C.innocuum; C. paraputrificum; C.perfringens* ***Coprococcus** comes; C. eutactus* ***Prevotella** copri* ***Roseburia** hominis; R. intestinalis; R. inulinvorans;* ***Ruminococcus** bromii; R. gnavus; R. obeum; R. torques*	
Chang et al. [119] Human	**YCFA**	Pre-incubation in blood culture bottles supplemented with 10% sheep blood and 10% rumen	Aero-intolerant bacteria ***Alistipes** shahii; A. onderdonkii,* ***Clostridium** bifermentans, C. innocuum, C. hiranonis, C. butiricum, C. hathewayi,* *C. bolteae, C. sporogenes,* ***Odoribacter** splanchnicus*	22% of species isolated increase: 16S RNA similarity 93–97% 3 new species isolated: ***Longicatena*** *caemuris* ***Bacillus alcalophilus Pseudogracilibacillus auburnensis***
Gotoh et al. [120] Microbial bank	**GAM**	NA	Aero-intolerant bacteria 72% of species of the top 56 species listed in the “human gut microbial gene catalogue” cultured in GAM	Isolated species in GAM: ***Anaerotruncus** colihominis,* ***Blautia** hansenii,* ***Clostridium** nexile, C. asparagiforme, C. scindens,* ***Coprococcus** comes* ***Roseburia** intestinalis* ***Ruminococcus** torques, R. lactaris, R. obeum, R. gnavus*.
Lagier et al. [121] 16-years-old male	**BHI**	Preincubation of the stool with lytic *E. coli* T1 and T4 phages	Non-fastidious aerobic and facultatively anaerobic bacteria	***Enterobacter****massiliensis* strain JC163T
Bailey and Coe [122] Rhesus Monkeys	**BHI**	NA	Non-fastidious aerobic and facultatively anaerobic bacteria	NA
	**EMB**	NA	Gram-negative aerobic and facultatively anaerobic bacteria	NA
	**LBS**	NA	Aerobic members of lactobacilli	***Lactobacillus*spp**.
Lei et al. [123] Female mice	**GMM**	NA	Gut aero-intolerant bacteria	
López-Moreno [117]	**BHI**	Supplemented with Obesogens: BPA, BPS	Anaerobic facultative Firmicutes	***Staphylococcus*, *Bacillus****amyloliquefaciens **group***, ***Streptococcus****salivarius*
López-Moreno [117]	**MRS**	Supplemented with Obesogens: BPA, BPS	*Lactobacillus*, Enterobacteria	***Latilactobacillus** sakei, **Enterococcus** faecium*

YCFA: yeast-extract-casein hydrolysate-fatty acids; GAM: gifu anaerobic medium; BHI: brain–heart infusion; EMB: eosin methylene blue; LBS: *Lactobacillus* selection; GMM: gut microbiota medium; MRS; Man, Rogosa and Sharpe; BPA: Bisphenol A; BPS: Bisphenol S. Genera and species in bold letters highlight the microorganisms to be considered as potential NGP to be searched, cultured and assayed for their anti-obesity modulation effects.

## Data Availability

The data presented in this study are available in the article or supplementary material.

## Abbreviations

MDC	Microbiota-disrupting chemicals
NGP	Next-generation probiotics
GIT	Gastrointestinal tract
PCOS	Polycystic ovary syndrome
FAO	Food and Agriculture Organization of the United Nations
WHO	World Health Organization
ICT	Interventional clinical trials
PCS	Preclinical studies
DC	Dendritic cells
IL	Interleukin
LPS	Lipopolysaccharide
TLR2	Toll-like receptor 2
TNF	Tumor necrosis factor
WGS	Whole genome sequencing
NGS	New-generation sequencing
AN	Anorexia nervosa
HC	Healthy control
HL	Hyperlipidemia
HT	Hypertension
LH	Lean healthy
MetS	Metabolic syndrome
MHNO	Metabolically healthy non-obese
MHO	Metabolically healthy obese
MUNO	Metabolically unhealthy non-obese
MUO	Metabolically unhealthy obese
NAFLD	Non-alcoholic fatty liver disease
NASH	Non-alcoholic steatohepatitis
OB	Obese
OBH	Obese healthy
OBT2D	Obese type 2 diabetes
OW	Overweight
RISK1	Patients with only one disease
RISK2	Patients with two disease
RISK3	Patients with three disease
SS	Simple steatosis
T1D	Type 1 diabetes
T2D	Type 2 diabetes
TSNO	Tsumura Suzuki obese diabetes mice
TSOD	Tsumura Suzuki non obesity mice
BPA	Bisphenol A
BPS	Bisphenol S
YCFA	Yeast-extract-casein hydrolysate-fatty acids
GAM	Gifu anaerobic medium
BHI	Brain–heart infusion
EMB	Eosin methylene blue
LBS	Lactobacillus selection
GMM	Gut microbiota medium
MRS	Man, Rogosa, and Sharpe
RNA	Ribonucleic acid
rRNA	Ribosomal ribonucleic acid
DNA	Deoxyribonucleic acid
OTU	Operational taxonomic unit
EU	European Union
Ph. Eur.	European Pharmacopoeia
US	United States
GRAS	Generally recognized as safe
FDA	Food and Drug Administration
EFSA	European Food Safety Authority
QPS	Qualified presumption of safety
EMA	European Medicines Agency
MHLW	Ministry of Health and Welfare
FOSHU	Food for specified health use
FUFOSE	Functional food science in Europe
SFDA	State Food and Drug Administration
DSHEA	Dietary Supplement Health and Education Act
BLA	Biologic license application
CFIA	The Canadian Food Inspection Agency
GMP	Good manufacturing practice
CFU	Colony-forming units
OA	Open access
